# Comparison of nutritional status and growth curves of children and adolescents in the city of Goiânia, Goiás: cross-sectional study

**DOI:** 10.1590/1516-3180.2022.0643.R1.010623

**Published:** 2023-08-04

**Authors:** Rafael Ribeiro Alves, Tadeu Baptista, Vitor Alves Marques, Weder Alves da Silva, Marcelo Henrique Silva, Douglas de Assis Teles Santos, Carlos Alexandre Vieira

**Affiliations:** I MSc. PhD Student, Postgraduate Program in Health Sciences, School of Medicine, Universidade Federal de Goiás (UFG) (UFG), Goiânia (GO), Brazil.; II PhD. Associate Professor, Physical Education Department, Universidade Federal do Rio Grande do Norte (UFRN), Natal (RN), Brazil.; III MSc. PhD Student, Postgraduate Program in Health Sciences, School of Medicine, Universidade Federal de Goiás, Goiânia (GO), Brazil.; IV MSc. PhD Student, Postgraduate Program in Human Movement and Rehabilitation, Universidade Evangélica de Goiás (UniEVANGÉLICA), Goiânia (GO), Brazil.; V MSc. PhD Student, Postgraduate Program in Health Sciences, School of Medicine, Universidade Federal de Goiás (UFG), Goiânia (GO), Brazil.; VI PhD. Associate Professor, College of Physical Education, Universidade Estadual da Bahia (UNEB), Teixeira de Freitas (BA), Brazil.; VII PhD. Associate Professor, College of Physical Education and Dance, Universidade Federal de Goiás (UFG), Goiânia (GO), Brazil.

**Keywords:** Growth charts, Nutritional status, Body mass index, Public health, Obesity, Body weights and measures, Body weight, Unified health system

## Abstract

**BACKGROUND:**

Nutritional status and growth curves can affect cognitive development, increase the risk of infection, and contribute to the development of chronic diseases. Its etiology is related to food, socioeconomic, and maternal conditions. However, to date, no data on these parameters exist in the state of Goiás, Brazil.

**OBJECTIVE:**

To compare the nutritional status and growth curves of children and adolescents in the city of Goiânia, Goiás, Brazil.

**DESIGN AND SETTING:**

This was a cross-sectional study. A total of 529 individuals were recruited from a primary health center in the municipality.

**METHODS:**

To assess nutritional status, the sample was divided into three categories: 3–4, 5–10, and 11–19 years, with z-score classification considering body mass index for age. The classification of growth curves was performed considering the median height values for age, assuming two references: (a) young Brazilian population and (b) one recommended for international use. The independent sample T-test was used to compare anthropometric variables.

**RESULTS:**

The results showed that the classification of eutrophics represents a predominant percentage between both sexes (men: 03–04 = 55.4%; 05–10 = 57.6%; 11–19 = 53.5 % and women: 03–04 = 53.5%; 05–10 = 63.9%; 11–19 = 56.9%), and growth curves showed differences in specific periods in both sexes.

**CONCLUSIONS:**

It can be concluded that children and adolescents from the city of Goiânia present as predominance the eutrophic nutritional status, followed by the risk of overweight, underweight, obesity, and malnutrition of both sexes.

## INTRODUCTION

The World Health Organization (WHO) considers children people aged between 0 and 10 years, and adolescents between 10 and 19 years of age.^1^ The prevalence of children and adolescents in Brazil is around 60 million people.^2^ In this age group, several important changes occur over the years, and for an adequate growth process, understanding the variables involved in this stage, such as the factors associated with malnutrition, overweight, and obesity, is essential.^3^

Malnutrition is a nutritional status directly related to infant morbidity and mortality, with a prevalence of up to 59% in certain regions such as Timor-Leste, Burundi, Niger, and Madagascar.^4^ This nutritional status can affect cognitive development, increase the risk of infections, and contribute to the development of chronic diseases such as diabetes, hypertension and coronary diseases.^5^ Its etiology is related to food, socioeconomic, maternal conditions, and health services^6^ and may even interfere with the negativity in the country's economy.^5^

Although the risks associated with malnutrition are concerning, paradoxically, overweight and obesity have become increasingly frequent among children and young people.^7,8^ This can contribute to the early development of chronic diseases, such as cardiovascular and metabolic,^9^ and may reduce the life expectancy of this population.^10^

In a study conducted by Gordon-Larsen et al.,^11^ evidence was found that the transition between adolescence and adulthood represents a period of risk for increased overweight and obesity regardless of sex. Therefore, monitoring nutritional status during the growth period can contribute to the prevention of these factors.

Growth curves enable the observation of the growth patterns of healthy individuals under environmental and social conditions favorable to their development. Thus, this instrument makes it possible to analyze and compare growth parameters in different regions of Brazil and other countries, observing the health condition of children and adolescents,^12^ not only the risks of malnutrition but also the prevalence of overweight and obesity in this population.^7^ These nutritional states may contribute to the early development of chronic diseases, such as cardiovascular and metabolic,^9^ and may reduce the life expectancy of this population.^10^

Growth curves are internationally accepted standards for observing differences between populations or subgroups in a given region with regard to the health condition of children and adolescents.^12^

Therefore, the control of nutritional status and physical growth can contribute to the prevention of diseases and the creation of government actions to improve the quality of life of the general population.

## OBJECTIVE

This study aimed to compare the nutritional statuses and growth curves of children and adolescents in the cities of Goiania and Goiás, Brazil.

## METHOD

To compare the nutritional status and growth curves, data were collected from a primary center of public health care in the city of Goiania, Brazil. Health centers are managed by the Brazilian Unified Health System in Goiania, Brazil. Data were collected between September and October 2011 from the medical records of individuals aged 3–19 years old.

A priori sample analysis revealed that to achieve a 0.5 effect size (ES) with a power of 0.95, a total of 210 participants would be necessary. Therefore, 529 participants were recruited to account for eventual attrition, which was approved by the Ethics Committee in Human Research, Universidade Federal de Goiás (CEP/CAAE:64091717.0.0000.5083), on March 9, 2017. All procedures were performed in accordance with the Declaration of Helsinki.

The sample was stratified into four categories for characterization: 3–5, 6–10, 11–15, and 16–19 years. Body mass and height were measured using a scale and stadiometer (Filizola PL200, São Paulo, Brazil) with accuracies of 100 g and 0.1 cm, respectively (Table 1).

**Table 1:** Characterization of the participants

Variable		n	Men	n	Women	P^*^
			Mean (DP)		Mean (DP)	
**Age (years)**						
	03–05	109	3.9 ± 0.9	86	4.0 ± 0.8	0.376
	06–10	69	7.8 ± 1.5	60	7.4 ± 1.5	0.207
	11–15	52	13.1 ± 1.5	43	12.6 ± 1.1	0.430
	16–19	35	17.6 ± 1.1	48	17.7 ± 1.2	0.585
**Body mass**						
	03–05	109	15.7 ± 3.5	86	15.6 ± 2.9	0.848
	06–10	69	24.9 ± 7.1	60	23.2 ± 6.6	0.221
	11–15	52	42.8 ± 14.6	43	42.9 ± 16.0	0.975
	16–19	35	66.0 ± 16.5	48	54.3 ± 9.8	0.003
**Stature**						
	03–05	109	1.0 ± 0.1	86	1.0 ± 0.1	0.926
	06–10	69	1.2 ± 0.1	60	1.2 ± 0.1	0.399
	11–15	52	1.5 ± 0.2	43	1.5 ± 0.2	0.525
	16–19	35	1.7 ± 0.1	48	1.6 ± 0.1	0.000
**Body mass index**						
	03–05	109	15.7 ± 1.8	86	15.3 ± 1.8	0.241
	06–10	69	16.2 ± 2.5	60	15.7 ± 2.3	0.502
	11–15	52	18.9 ± 4.0	43	19.6 ± 4.8	0.432
	16–19	35	22.7 ± 4.5	48	21.8 ± 3.0	0.900

The body mass index (BMI) was calculated by dividing the weight in kilograms by height square into meters. For nutritional status assessment, the sample was divided into three age categories (2007):^13^ 3–4 (3 ≥ age ≤ 4 years), 5–10 (5 ≥ age ≤ 10 years), 11–19 (11 ≥ age ≤ 19 years); as a classification parameter, the score-z was adopted considering BMI for age (Table 2).

**Table 2:** Classification of nutritional status of males based on the World Health Organization (2007)

Age (year)	BMI-stature						
	n	ST	TH	ET	OW	OB	SB
03–04	74	1.4%	5.4%	55.4%	20.3%	12.2%	5.4%
05–10	60	5%	7.5%	57.6%	22.5%	6.3%	1.3%
11–19	52	3.8%	13.5%	53.8%	17.3%	7.7%	3.8%

BMI = body mass index; ST = severe thinness; TH = thinness; ET = eutrophy; OW = overweight; OB = obesity; SB = severe obesity.

**Figure 1:**
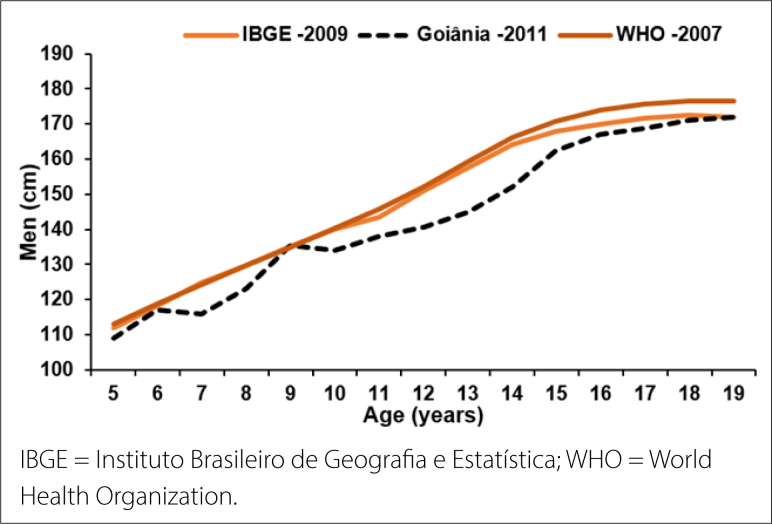
Comparison of the growth curves of men in Goiânia with Instituto Brasileiro de Geografia e Estatística and World Health Organization data.

**Figure 2:**
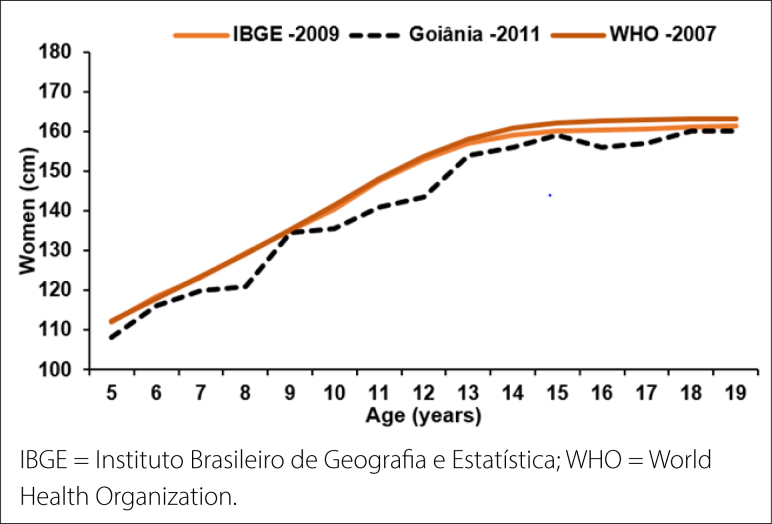
Comparison of women's growth curves in Goiânia with Instituto Brasileiro de Geografia e Estatística and World Health Organization data.

The classification of physical growth curves (Figures 1 and 2) was performed by graphical comparison of the median values of height for age, assuming two references: (a) the Brazilian young population (Instituto Brasileiro de Geografia e Estatística [IBGE])^14^ and (b) recommended for international use by the World Health organization.^13^

The normality of age, body mass, height, and body mass index data was analyzed using the Kolmogorov–Smirnov test. For comparison between variables, the t-test was used for independent samples for parametric data and the Mann–Whitney test for non-parametric data. Statistical significance was set at P < 0.05.

## RESULTS

A total of 253 females (9.55 ± 5.36 years, 31.50 ± 18.06 kg, 1.28 ± 0.25 m) and 276 males (8.42 ± 4.96 years, 29.66 ± 19.43 kg, 1.24 ± 0.28 m) were evaluated.

Groups aged between 16 and 19 years (16 ≤ 19 years) demonstrated significant difference in body mass (54.3 ± 9.8 and 66.00 ± 16.5) (P = 0.003), as well as in height (1.7 ± 0.1 and 1.6 ± 0.1) (P = 0.000), between men and women, respectively (Table 1). However, no significant differences were found in the other variables.

The classification of nutritional status using the Z-score (Tables 2 and 3) showed that eutrophication represents a higher percentage among groups of men (03–04 years = 55.4%; 05–10 years = 57.6%; 11–19 years = 53.8%) and women (03–04 years = 53.5%; 05–10 years = 63.9%; and 11–19 years = 56.9%). Despite the increased overweight in men (17.3%) compared with women (12.1%) in the age group between 11 and 19 years, overweight and obesity have higher percentages in women (13.8% and 5.2%, respectively), when compared with men (7.7% and 3.8%, respectively). In this context, a higher percentage of overweight and obesity was demonstrated in men only between 3 and 4 years (12.2% and 5.4%, respectively) when compared with women (8.6% and 1.7%, respectively).

**Table 3:** Classification of nutritional status of females based on the World Health Organization (2007)

Age (year)	BMI-stature						
	n	ST	TH	ET	OW	OB	SB
03–04	58	1.7%	12.1%	53.5%	22.4%	8.6%	1.7%
05–10	60	1.6%	8.2%	63.9%	23.0%	3.3%	0%
11–19	58	3.4%	8.6%	56.9%	12.1%	13.8%	5.2%

BMI = body mass index; ST = severe thinness; TH = thinness; ET = eutrophy; OW = overweight; OB = obesity; SB = severe obesity.

However, thinness had a higher prevalence percentage in women aged 3–4 years compared with men (12.1% and 5.4%, respectively); In addition, in groups 11 and 19 years, men had higher percentages of thinness than women (13.5% and 8.6%, respectively).

The growth curves between men and women (Figures 1 and 2) demonstrated differences between the sexes at 7 and 9 years, as well as between 15 and 17 years, with higher values for men. However, a decline in the curve for men aged 13 and 15 years was demonstrated when compared with the IBGE and WHO data.

## DISCUSSION

This study aimed to verify the nutritional status and growth curve of children and adolescents in the city of Goiânia, Goiás. The results showed that male adolescents aged 16–19 years had greater heights and body masses than female adolescents. Regarding nutritional status, male and female children and adolescents had a higher prevalence of eutrophy; however, overweight and obesity were observed in all age groups. In addition, we found a significant difference between sexes in height and body mass in the age group of 16–19 years (P = 0.00 and P = 0.00, respectively).

Additionally, body mass, height, and nutritional status are related. In a systematic review by Junior et al.^15^ that analyzed the influence of these two factors on body fat in individuals aged 2–19 years, the results demonstrated a positive correlation between these factors and body fat in both sexes (men, r = 0.975; women, r = 0.947). This demonstrates the importance of monitoring growth curves during adolescence as a form of care for adults with obesity.

Regarding the growth curve, at the age of 19 years, a similar curve was verified among men in our study with IBGE data from 2009 (1.70 cm); however, the curve was lower than the WHO in 2007 (1.80 cm). However, women presented a lower growth curve (1.55 cm) than that of the IBGE (1.58 cm) and WHO (1.60 cm) criteria. This demonstrates that Goiânia adolescents present a shorter stature compared with the two references adopted. This is justified by the average height of the population of Goiania, which is 1.75 cm for men and 1.78 cm for women, below those of the IBGE and WHO.^13,14^

Regarding the findings on the classification of nutritional status, the results showed low percentages of obese adolescents with overweight in men (3.8% and 7.7%) as well as in women (5.2% and 13.8%), which do not corroborate with those of other regions in the country. Geremia et al.,^16^ estimated the prevalence of overweight and obesity in adolescents with a mean age of 12.45 ± 1.49 years in the city of Bento Gonçalves, in the interior of the state of Rio Grande do Sul, Brazil. Male adolescents have levels of overweight and obesity of 16.3% and 12.2%, respectively. However, female adolescents have levels of overweight and obesity of 16.2 and 5.5%, respectively. Possibly, the factors inherent in the higher percentage of obese and overweight individuals in the south of the country may be cultural influences, as some of the populations are Italian and German immigrants. Another factor contributing to the low rate of obesity and overweight among adolescents is the level of physical activity. In our study, adolescents in public education in Goiás, children, and adolescents who attend public schools have a higher level of physical activity than those who attend private education.^8^

However, when we compared our results with those of other Latin American countries, our results corroborate with the study of Atalah et al.^17^ in Chile referring to the “eutrophic” nutritional status of adolescent men (55.4% and 62.18%, respectively) and overweight (20.3% and 15.83%, respectively). Additionally, the adolescent men in our study had a lower percentage of obesity (3.8%) compared with those in Chile (16.6%), which may be associated with the eating factors of the country; in addition, this divergence may have been influenced by the economic level and social differences, in which the higher the socioeconomic level and the lower the inequality, the higher the obesity,^13^ which may justify the percentage results of obesity similar to the study (5.4%) reported by Capanzana et al.^18^ on the Philippines, as this country has low social status, coupled with natural disasters, such as tsunamis, along with low investment in public policies.

In this context, despite similar results in other nutritional statuses, the study of Camarinha Graça e Nogueira,^19^ conducted in Portugal, also reported percentage results of higher obesity (6.3%) than that in our study, demonstrating that countries with higher human development index (HDI) appear to favor the development of obesity, which is an important finding for future research.

Interestingly, we found results contrary to this hypothesis in the study by Ubesie et al.^20^ in Africa, more specifically in southwestern Nigeria. Sixty-five percent of the children and adolescents were eutrophic, and 2% were underweight. These values resemble the nutritional status of young people in the city of Goiânia with the same age group: 63.9% eutrophic and 1.6% low weight. However, although Nigeria has a smaller HDI than Brazil, southeastern Nigeria has a better level of existence than the rest of the country, thus offering better living conditions for children and adolescents in this region.^21^ Similar to the Nigerian study, children and adolescents also have low socioeconomic levels, which explains 1.6% of the population is underweight.^21^

However, HDI also appears to reflect the growth curve, and the study of Gomez-Campos et al.^22^ analyzed the growth curve of children in Chile with a mean age of 11 years, noting a height greater than that of Brazilian children in both sexes (men 1.45 cm and women 1.35 cm). However, when we compared our findings with countries, such as East Timor, which has high levels of malnutrition and environmental disasters, as well as military conflicts, we found lower results than those found in our study in male adolescents (1.40 versus 1.60 cm) and females (1.30 versus 1.50 cm), as well as in that of the WHO (1.70 and 1.60 cm, respectively). This shows that the socioeconomic conditions in Brazil are better than those in some African and Asian countries.

In this sense, we find differences in the growth curve of our study compared with those of other countries in Europe. Riedlová et al.^23^ analyzed 960 male children born in the Czech Republic aged 12 years. The mean height was 1.81 cm, which is higher than our findings (1.40 cm), as well as that of the WHO (1.79 cm). This difference may be related to the best living and feeding conditions of European children compared with those of Brazilian children.^21^ In addition, genetic influences may also influence this variable.^24^

Cultural aspects can also influence the growth curve as shown in a study conducted by Bahchachi et al.^25^ who analyzed the growth curve of 7,772 Algerian adolescents of both sexes. The results showed that female adolescents had an average of 1.57 cm of height, whereas male adolescents had an average of 1.75 cm. The Algerian study results are higher than that of the Goiânia male adolescents (1.70 cm); however, when compared with the females, the growth curve of Brazilian adolescents were higher (1.60 cm). In addition to Algeria, which had a high HDI (0.754), such differences between sexes may be related to cultural aspects. Algeria is a Muslim country where an explicit difference exists between the sexes, in which female children and adolescents are oriented to perform domestic services and take care of the family, while the opposite sex has more opportunities to develop their physical aspects, such as physical exercise and better feeding.^21^ This demonstrates how much the physical and social environments influence nutritional status.^3^

Additionally, the comparison of growth curves between different regions of Brazil showed similar results between the cities of Campinas and Goiânia. Campos et al.^26^ analyzed the growth curve of children and adolescents in the city of Campinas (SP). The results were similar in adolescents of both sexes aged 18 years: women presented an average height of 1.55 cm, whereas men, 1.70 cm. These values corroborate the results of our study, demonstrating that despite children and adolescents presenting a growth curve lower than that of the IBGE, it resembles other Brazilian cities.

Monego e Jardim^27^ conducted a population-based study with 3,169 students in eastern Goiânia and identified overweight and obesity levels of 10.8% and 5.3% among male students and 11.3% and 4.5% among girls, with an average of 10.7 years (10.72 for boys and 10.76 for girls), presenting higher levels of overweight and obesity when compared with the results of this study for the age group of 5 to 10 years. The study by Monego e Jardim in 2006 was performed using data from students in 2001. Our study used data from 2011. In 10 years, the percentage of children and adolescents who were obese decreased, which is due to an increase in the practice of activity mainly in schools and an improvement in the population's socioeconomic levels.^3^

Therefore, the differences in nutritional status and growth curve are directly related to the culture of countries, socioeconomic conditions, and public policies that each region implement within its own state.^28^ Particularly at the end of adolescence and early adulthood, a relationship exists between the response to adverse events and weight changes.^29^ One manner to improve the growth curve related to body mass may be the practice of physical exercise. Adolescents who practice physical exercise have adequate control of body weight; however, inadequate lifestyles can impair this development.^30^ Our research has a significant sample and is an original study. Not research has yet evaluated the growth curve of children and adolescents in both sexes in the city of Goiânia. As a limitation, we did not assess the socioeconomic levels and levels of physical activity. These results can contribute to the creation of public policies in the city of Goiânia and the region, which aim to reduce the risk of overweight and obesity, in addition to improving aspects related to growth curves and consequently economic, social and cultural development, through the investment of public policies in the sectors inherent to these aspects.

## CONCLUSION

Children and adolescents in the city of Goiânia present a predominantly eutrophic nutritional status, followed by the risk of overweight, underweight, obesity, and malnutrition in both sexes. A trend of increasing BMI over time exists. Additionally, the growth curve was lower than those of the WHO and IBGE levels. Despite the optimistic results regarding the percentage of eutrophication, the risk of being overweight is high among men and women, which corroborates with some epidemiological studies that demonstrated a prevalence relationship of overweight in developed regions when compared with less developed regions, demonstrating that improving access to information inherent to body mass control and general health is required.

Additionally, further studies should be conducted in other regions of the state and country to verify the aspects inherent to the development of the population, which directly reflects national development.

## References

[1] Organización de las Naciones Unidas para la Agricultura y la
Alimentación/Organización Mundial de la Salud (2014). Segunda Conferencia Internacional sobre Nutrición Roma, 19-21 de
noviembre de 2014. Documento final de la Conferencia: Declaración de Roma
sobre la Nutrición.

[2] Instituto Brasileiro de Geografia e Estatística - IBGE Downloads.

[3] Malta DC, Andrade SC, Claro RM, Bernal RTI, Monteiro CA (2014). Trends in prevalence of overweight and obesity in adults in 26
Brazilian state capitals and the Federal District from 2006 to
2012. Rev Bras Epidemiol.

[4] United Nations Chilsdren's Fund (2013). Improving child nutrition: The achievable imperative for global
progress.

[5] Victora CG, Adair L, Fall C (2008). Maternal and child undernutrition: consequences for adult health
and human capital. Lancet.

[6] Souza OF de, Benício MHD, Castro TG de, Muniz PT, Cardoso MA (2012). Desnutrição em crianças menores de 60 meses em dois municípios no
Estado do Acre: prevalência e fatores associados. Rev Bras Epidemiol.

[7] Hossain P, Kawar B, El Nahas M (2007). Obesity and diabetes in the developing world--a growing
challenge. N Engl J Med.

[8] Mondini L, Levy RB, Saldiva SR (2007). Prevalência de sobrepeso e fatores associados em crianças
ingressantes no ensino fundamental em um município da região metropolitana
de São Paulo, Brasil [Overweight, obesity and associated factors in first
grade schoolchildren in a city of the metropolitan region of São Paulo,
Brazil]. Cad Saude Publica.

[9] Campbell I (2003). The obesity epidemic: can we turn the tide?. Heart.

[10] Gouveia ER, Freitas DL, Maia JA (2007). Atividade física, aptidão e sobrepeso em crianças e adolescentes:
“o estudo de crescimento da Madeira”. Rev Bras Educ Fís Esporte.

[11] Gordon-Larsen P, Adair LS, Nelson MC, Popkin BM (2004). Five-year obesity incidence in the transition period between
adolescence and adulthood: the National Longitudinal Study of Adolescent
Health. Am J Clin Nutr.

[12] Silveira FJF, Lamounier JA (2009). Avaliação nutricional de crianças do Vale do Alto Jequitinhonha
com a utilização das novas curvas de crescimento do NCHS e da
OMS. Rev Paul Pediatr.

[13] WHO (2007). Growth reference data for 5-19 years.

[14] Instituto Brasileiro de Geografia e estatística - IBGE (2010). Pesquisa de orçamentos familiares 2008-2009: antropometria e estado
nutricional de crianças, adolescentes e adultos no Brasil.

[15] Alves CA, Mocellin MC, Gonçalves ECA, Silva DA, Trindade EB (2017). Anthropometric indicators as body fat discriminators in children
and adolescents: a systematic review and meta-analysis. Adv Nutr.

[16] Geremia R, Cimadon HM, de Souza WB, Pellanda LC (2015). Childhood overweight and obesity in a region of Italian
immigration in Southern Brazil: a cross-sectional study. Ital J Pediatr.

[17] Atalah E, Cordero M, Guerra ME (2014). Monitoreo de los indicadores del Programa “Chile Crece Contigo”
2008-2011 [Monitoring indicators of the program “Chile Grows with You”
2008-2011]. Rev Chil Pediatr.

[18] Capanzana MV, Aguila DV, Gironella GMP, Montecillo KV (2018). Nutritional status of children ages 0-5 and 5-10 years old in
households headed by fisherfolks in the Philippines. Arch Public Health.

[19] Camarinha B, Graça P, Nogueira PJ (2016). A prevalência de pré-obesidade/obesidade nas crianças do ensino
pré-escolar e escolar na autarquia de Vila Nova de Gaia, Portugal
[Prevalence of pre-obesity/obesity in pre and basic school children at Vila
Nova de Gaia, Portugal]. Acta Med Port.

[20] Ilechukwu G, Ilechukwu C, Ubesie A (2014). Relationship between nutritional status and intensity of common
intestinal helminths among children in enugu, South-East
Nigeria. Ann Med Health Sci Res.

[21] WHO (2013). The world health report 2013: Research for Universal Health
Coverage.

[22] Gomez-Campos R, Arruda M, Luarte-Rocha C (2016). Enfoque teórico del crecimiento físico de niños y
adolescentes. Rev Esp Nutr Hum Diet.

[23] Riedlová J, Vignerová J, Paulová M (2017). Body parameters of Czech breastfed children compared to the Czech
references and WHO growth standards. Ann Hum Biol.

[24] Arı Yuca S, Cesur Y, Kurtoğlu S, Mazıcıoğlu MM, Cimbek EA (2014). Growth patterns of children of same geographic background reared
in different environments. J Clin Res Pediatr Endocrinol.

[25] Bahchachi N, Dahel-Mekhancha CC, Rolland-Cachera MF (2017). Courbes de l'indice de masse corporelle d'enfants et adolescents
algériens (6–18 ans) [Body mass index charts of Algerian children and
adolescents (6–18 years)]. Arch Pediatr.

[26] Campos RG, de Arruda M, Hespanhol JE (2015). Referencial values for the physical growth of school children and
adolescents in Campinas, Brazil. Ann Hum Biol.

[27] Monego ET, Jardim PC (2006). Determinantes de risco para doenças cardiovasculares em escolares
[Determinants of risk of cardiovascular diseases in
schoolchildren]. Arq Bras Cardiol.

[28] Silva DA, Pelegrini A, Petroski EL, Gaya AC (2010). Comparison between the growth of Brazilian children and
adolescents and the reference growth charts: data from a Brazilian
project. J Pediatr (Rio J).

[29] Elsenburg LK, Smidt N, Liefbroer AC (2017). The longitudinal relation between accumulation of adverse life
events and body mass index from early adolescence to young
adulthood. Psychosom Med.

[30] Carvalho EG, Matos LM, Cavalcante ACM, Almeida JZ (2013). Perfil nutricional de adolescentes praticantes de exercício
resistido. Rev Bras Promoc Saude, Fortaleza.

